# Exploration of Individual and System-Level Well-being Initiatives at an Academic Surgical Residency Program: A Mixed-Methods Study

**DOI:** 10.1001/jamanetworkopen.2020.32676

**Published:** 2021-01-06

**Authors:** Carter C. Lebares, Anya L. Greenberg, Nancy L. Ascher, Kevin L. Delucchi, Linda M. Reilly, Marieke van der Schaaf, Fredrik Baathe, Patricia O’Sullivan, Karin Isaksson Rø

**Affiliations:** 1 Department of Surgery, University of California, San Francisco; 2 Department of Psychiatry, University of California, San Francisco; 3 Center for Research and Development of Health Professions Education, University Medical Center Utrecht, Utrecht, the Netherlands; 4 Institute of Care and Health Services, University of Gothenburg, Gothenburg, Sweden; 5 Institute of Stress Medicine, Gothenburg, Sweden; 6 Institute for the Studies of the Medical Profession, University of Oslo, Oslo, Norway

## Abstract

**Importance:**

Physician well-being is a critical component of sustainable health care. There are few data on the effects of multilevel well-being programs nor a clear understanding of where and how to target resources.

**Objective:**

To inform the design of future well-being interventions by exploring individual and workplace factors associated with surgical trainees’ well-being, differences by gender identity, and end-user perceptions of these initiatives.

**Design, Setting, and Participants:**

This mixed-methods study among surgical trainees within a single US academic surgical department included a questionnaire in January 2019 (98 participants, including general surgery residents and clinical fellows) and a focus group (9 participants, all clinical residents who recently completed their third postgraduate year [PGY 3]) in July 2019. Participants self-reported gender (man, woman, nonbinary).

**Exposures:**

Individual and organizational-level initiatives, including mindfulness-based affective regulation training (via Enhanced Stress Resilience Training), advanced scheduling of time off, wellness half-days, and the creation of a resident-driven well-being committee.

**Main Outcomes and Measures:**

Well-being was explored using validated measures of psychosocial risk (emotional exhaustion, depersonalization, perceived stress, depressive symptoms, alcohol use, languishing, anxiety, high psychological demand) and resilience (mindfulness, social support, flourishing) factors. End-user perceptions were assessed through open-ended responses and a formal focus group.

**Results:**

Of 98 participants surveyed, 64 responded (response rate, 65%), of whom 35 (55%) were women. Women vs men trainees were significantly more likely to report high depersonalization (odds ratio [OR], 5.50; 95% CI, 1.38-21.85) and less likely to report high mindfulness tendencies (OR, 0.17; 95% CI, 0.05-0.53). Open-ended responses highlighted time and priorities as the greatest barriers to using well-being resources. Focus group findings reflected Job Demand–Resource theory tenets, revealing the value of individual-level interventions to provide coping skills, the benefit of advance scheduling of time off for maintaining personal support resources, the importance of work quality rather than quantity, and the demoralizing effect of inefficient or nonresponsive systems.

**Conclusions and Relevance:**

In this study, surgical trainees indicated that multilevel well-being programs would benefit them, but tailoring these initiatives to individual needs and specific workplace elements is critical to maximizing intervention effects.

## Key Points

**Question:**

Are there targetable individual characteristics and workplace elements that are associated with surgical resident well-being, and do these differ by gender identity?

**Findings:**

In this mixed-methods study of 98 US surgical trainees, women surgical residents were significantly more likely to report high depersonalization and lower mindfulness tendencies compared with men trainees. Scheduling conflicts and organizational priorities emerged as the greatest barriers to using well-being resources; training in affective regulation skills, advance scheduling of time off, attention to work quality (vs quantity), and avenues of recourse for rectifying inefficient systems were cited as key components of an effective and holistic well-being program.

**Meaning:**

In this study, participants indicated that multilevel and holistic well-being programs would benefit surgical trainees, but tailoring them to address individual characteristics and workplace elements is critical.

## Teaser

This mixed-methods study explores individual and workplace factors that are associated with surgical trainees’ well-being, differences by gender identity, and end-user perceptions of experience.

## Introduction

Physician well-being is a critical component of sustainable health care.^1^ Its absence affects the personal and professional lives^2^ of surgeons and trainees, with resultant distress in the form of burnout,^3^ depression,^4^ alcohol abuse,^4,5^ and attrition, which carry a profound economic impact.^6^

While risk factors have become clearer, factors that enhance physician well-being remain poorly understood.^3,4,7^ Increasing evidence suggests that both individual and organizational-level interventions are necessary^3,8,9^ but likely need tailoring to meet the needs of diverse individuals, groups, and settings.^10,11,12^ To date, there are few data on multilevel well-being programs among surgical trainees, nor a nuanced understanding of where and how to tailor interventions to optimize the use of limited resources.

To address this gap, we drew on both Broaden-and-Build^13^ and Job Demand–Resource^14^ theories to conceive of ways in which individual and workplace factors might be associated with surgical trainee well-being. We then surveyed surgical trainees in the University of California San Francisco (UCSF) Department of Surgery (DOS) using published measures of individual and occupational risk and resilience factors. We additionally held a focus group exploring surgical residents’ perceptions of existing well-being initiatives at our institution and aspects of their work experience. Our goal was to help to inform the design of future multilevel well-being initiatives by exploring individual and workplace factors associated with well-being, evaluating differences by gender identity, and assessing end-user experience.

## Methods

### Study Design and Population

We conducted a mixed-methods study comprised of 2 components. The first component was a questionnaire administered in January 2019, after 3 consecutive years of Enhanced Stress Resilience Training (ESRT) training for surgical trainees in postgraduate year (PGY) 1 and 1 year of organizational-level well-being initiatives for all DOS PGY levels. The second component was a focus group conducted in July 2019, comprised of volunteer surgical residents who had recently completed their PGY 3. Respondents to the questionnaire and focus group participants were asked to self-report their gender identities (man, woman, nonbinary) as part of their participation in our study.

All 83 residents (including those engaged in clinical duties and those conducting research as part of our academic general surgery residency program) and 15 clinical fellows in the UCSF DOS received the online questionnaire. Because of stigma surrounding mental illness in medicine,^15,16,17^ respondents were guaranteed anonymity. The American Association for Public Opinion Research (AAPOR) standard definitions were followed for calculating survey response rate.

The focus group involved off-site dinner but no compensation. A semistructured script was used, expanding on questions from the questionnaire with elaboration prompts. By participant request, no recording was made, but extensive field notes were taken. The Standards for Reporting Qualitative Research (SRQR) reporting guideline was followed. The study was approved by UCSF’s institutional review board, and informed consent was obtained from participants.

### Individual and Organizational Well-being Initiatives

In the UCSF DOS, resident well-being interventions began in response to data reflecting a high prevalence of burnout and distress. In 2016, we designed ESRT, an individually based intervention tailored to surgeons that teaches cognitive skills for affective regulation (ie, self-awareness, perspective taking, and control of emotional reactions to thoughts and events). We tested ESRT in 2 randomized clinical trials with a total of 65 surgery trainees in PGY 1 (21 categorical trainees) between January 2016 and December 2018 and reported feasibility^18^; improvements to cognitive function, burnout, mindfulness, and physiologic stress^19^; and activation of neural substrates associated with top-down executive function and interoception (ie, awareness of one’s own thoughts, sensations, and emotions).^19,20^

In 2018, multiple organizational initiatives were introduced: an administrative chief resident role (allowing for advanced scheduling of resident time off) and representation on education committees (influencing organizational-level changes). Results included biannual wellness half-days, a resident research fund, personal finance seminars, social gatherings, and healthy workplace food.

### Well-being Measures

Scales and measures used in our prior work, related to performance and/or distress in surgeons,^4,5,21,22^ were used and scored according to published methods. These include the Maslach Burnout Inventory, a validated 2-item screening that assesses emotional exhaustion and depersonalization^23^; Cohen Perceived Stress Scale,^4^ a criterion-standard measure of stress, with normative data for men and women aged 18 to 34 years, used to determine high cutoffs (ie, published mean + [0.5 × SD]) of at least 19.825 for women and at least 17.29 for men^24^; the Patient Health Questionnaire–2,^4^ a rigorously evaluated and validated depression screening tool,^25^ with a cutoff of 3 correlated with increased use of clinical resources^26^; the Alcohol Use Disorders Identification Test,^4^ a World Health Organization screening tool validated in large general population and veteran samples, used to assess alcohol misuse and abuse with cutoffs for gender^4^; and the Spielberger State-Trait Anxiety Index,^4^ a measure of subjective feelings (eg, apprehension, tension) and autonomic arousal^27,28,29,30,31^ correlated with state anxiety, used with surgical trainees in real-life and simulated trauma^28,29,30,31^ scenarios, with a score of at least 40 used as the cutoff for high anxiety in other studies.^30,31^ Also included were measures exploring the presence of wellness (not just pathology) and resiliency (characterized by high positive emotions, acceptance or nonreactivity to stressors, and connectedness or high social support), as defined by seminal works in the field of resilience science.^13,32^

Finally, we explored elements of occupational strain that might reveal areas for focused organizational-level change. Respectively, these measures included: the Cognitive Affective Mindfulness Scale–Revised (CAMS-R), a measure of both dispositional and trained mindfulness, defined as a tendency to use the specific cognitive skills of interoception (attention to one’s thoughts, feelings, and sensations), present focus (as opposed to focusing on the past or future), open awareness (of real-time situations and events), and acceptance (or affective regulation/nonreactivity),^4^ with a calculated global score and high cutoff of at least 31 used in others’ work^33^; the Mental Health Continuum–Short Form, a 14-item measure of individual social, emotional, and psychological well-being, with scores grouped by domain to categorize flourishing (high positive functioning, high positive emotions) or languishing (low positive functioning, low positive emotions)^34^ and lower scores correlated with the risk of developing depression, increased health care utilization, and self-reported academic impairment^26^; and the Demand-Control-Support Questionnaire, a 17-item measure of job strain with subdomains for psychological demand, control, and social support.^35^ Only demand and social support (12 items) were assessed in this study. High subdomain cutoffs were defined by convention as scores in the upper tertile of possible scores.^36^

### End-User Experience

We explored end-user experience through evaluation of responses to 2 open-ended survey questions and thematic analysis of qualitative content from a 3-hour focus group (moderated by C.C.L., P.O., and N.L.A., surgical and medical education faculty). The focus group followed a semistructured script exploring resident perceptions of influences, personal changes, and residency program elements, with 9 volunteer surgical trainees in PGY 3.

### Statistical Analysis

Survey response data were converted to numeric scores and used as continuous variables of psychosocial risk and resilience factors. Binary categorical variables were established for select factors (cutoffs discussed previously) to determine the prevalence of high levels of these factors, with odds ratios (ORs) created to assess gender associations. Because distributions of several factors were skewed and discrete, the nonparametric measure, Kendall τ-b, was used to estimate the association among the different risk and resiliency factors, given that this test is not influenced by extreme measures. All tests were 2-tailed, with significance set at *P* < .05. All computations were performed with SAS version 9.4 (SAS Institute) by a senior biostatistician (K.L.D.).

The open-ended survey responses were compiled. Notes from the focus group were input into a spreadsheet and analyzed using inductive and deductive thematic analysis techniques^37^ following published guidelines and a 6-step approach.^38^ Codes, concepts, and themes were iteratively reviewed, refined, discussed, and described in the context of past work^18^ and current aims (by C.C.L. and A.L.G.). Qualitative results are reported according to SRQR^39^ guidelines.

## Results

### Respondents and Participants

Among the 98 DOS trainee survey recipients (83 residents and 15 clinical fellows), 64 responded, yielding a survey response rate of 65% (Table 1). No participants indicated a nonbinary gender identity. Of the 64 survey respondents, 35 (55%) were women, 54 (84%) were residents (40 [62%] engaged in clinical duties; 14 [22%] conducting research), and 10 (16%) were clinical fellows. Focus group participants were 9 volunteer categorical residents who recently completed their PGY 3 (5 [56%] women; 5 [56%] ESRT past participants).

**Table 1.** Demographic Characteristics of Survey Respondents

Characteristic	Survey respondents, No. (%) (N = 64)^a^
DOS residents	
Total	54 (84)
PGY 1	14 (22)
PGY 2	7 (11)
PGY 3	8 (13)
PGY 4	6 (9)
PGY 5	5 (8)
Research	14 (22)
DOS clinical fellows	10 (16)
Gender, No. (%)	
Men	29 (45)
Women	35 (55)

Abbreviations: DOS, Department of Surgery; PGY, postgraduate year.

^a^ There were a total of 98 trainees, 83 (85%) of whom were residents and 15 (15%) of whom were clinical fellows.

### Differences by Gender Identity

As shown in Table 2, women trainees were significantly more likely to report high depersonalization (OR, 5.50; 95% CI, 1.38-21.85) and less likely to report high mindfulness tendencies (OR, 0.17; 95% CI, 0.05-0.53) compared with men trainees. Gender differences were not significant for other factors.

**Table 2.** Prevalence of Risk and Resilience Factors for Surgical Trainees, By Gender

Psychosocial factor	Participants, No. (%)			OR (95% CI)
	All	Men	Women	
Risk				
High emotional exhaustion	18 (30)	4 (16)	14 (40)	3.50 (0.99-12.40)
High depersonalization	18 (30)	3 (12)	15 (43)	5.50 (1.38-21.85)^a^
High perceived stress	26 (45)	10 (43)	16 (46)	1.09 (0.38-3.16)
Depressive symptoms	5 (9)	2 (9)	3 (9)	1.00 (0.15-6.53)
Alcohol misuse or abuse	34 (53)	13 (45)	21 (60)	1.85 (0.68-5.00)
Alcohol abuse	16 (25)	4 (14)	12 (34)	3.26 (0.92-11.56)
Languishing	5 (8)	1 (4)	4 (11)	2.97 (0.31-28.33)
High psychological demand at work	45 (76)	18 (75)	27 (77)	1.13 (0.33-3.79)
Resilience				
High mindfulness	22 (37)	15 (60)	7 (20)	0.17 (0.05-0.53)^a^
Flourishing	38 (64)	19 (79)	19 (54)	0.31 (0.10-1.03)
High social support at work	40 (75)	19 (79)	21 (71)	0.66 (0.19-2.25)

Abbreviation: OR, odds ratio.

^a^ *P* < .05.

### Associations Between Risk and Resilience Factors

**Figure 1.**
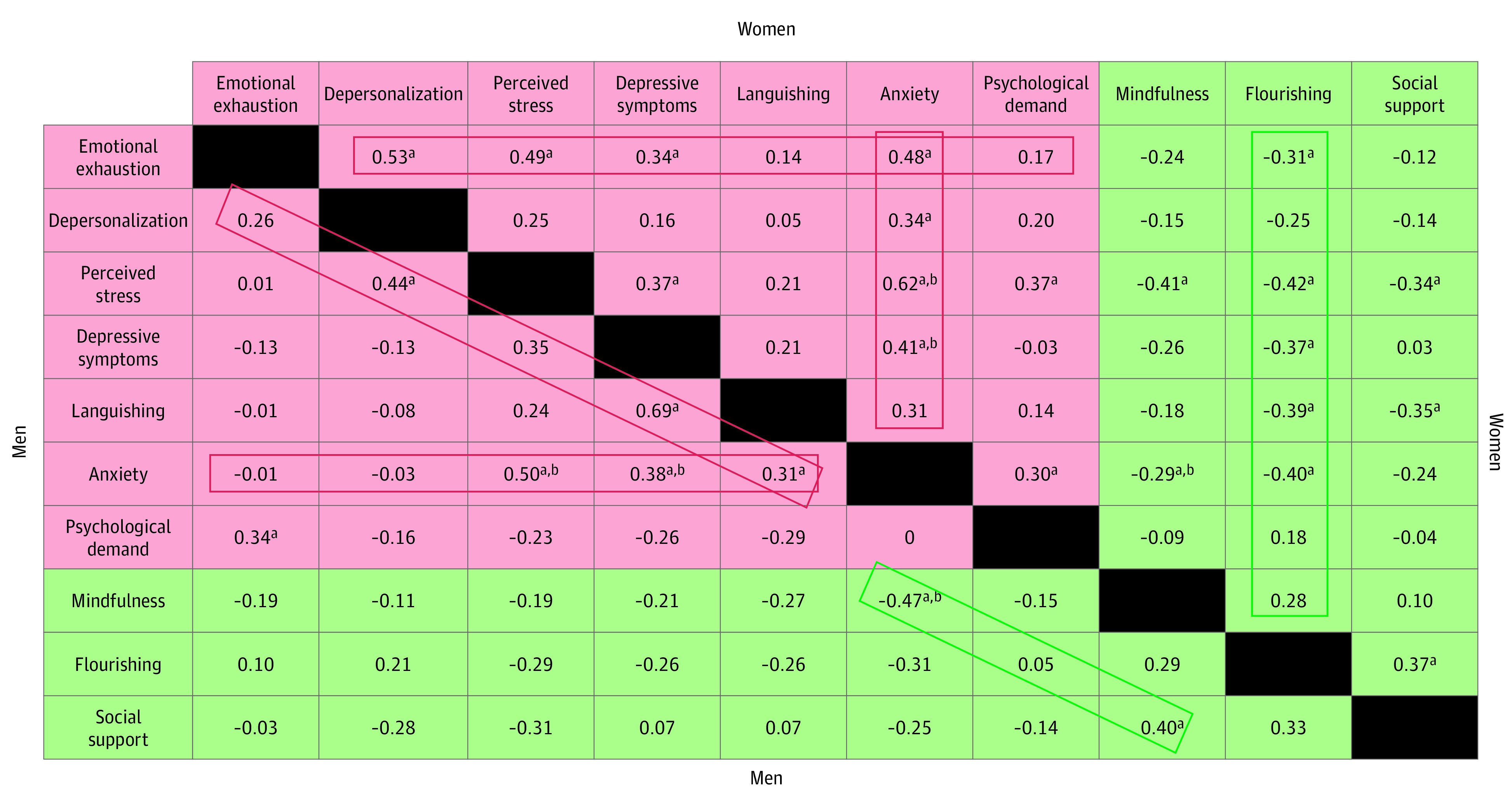
Correlation Patterns Between Risk and Resilience Factors by Gender Identity Red boxes indicate patterns in individual and workplace risk factor correlation; green boxes, patterns in individual and workplace resiliency factor correlation. ^a^*P* < .05. ^b^Statistically significant finding applies to both genders.

Individual and occupational risk and resilience factors by gender (Figure 1) revealed patterns and trends that suggest both shared and unique associations with well-being. Among women, factors influencing psychological risk were suggested by significant positive correlations between negative affect (such as depressive symptoms, high perceived stress, and anxiety), burnout (emotional exhaustion and depersonalization), and high workplace psychological demand (eg, depressive symptoms and emotional exhaustion, *r* = 0.34; perceived stress and workplace demand, *r* = 0.37). Factors influencing psychological resilience among women were suggested by significant positive correlations between positive affect (such as low perceived stress and flourishing), mindfulness, and high workplace social support (eg, flourishing and social support, *r* = 0.37). Overall, the state of flourishing among women trainees had a significant positive correlation with positive affect (eg, lower stress, depressive symptoms, languishing and anxiety), lower burnout (specifically emotional exhaustion), and high workplace social support.

Among men, factors influencing psychological risk were suggested by significant positive correlations between negative affect (such as depressive symptoms, languishing, high perceived stress, and anxiety), burnout (emotional exhaustion and depersonalization), and high workplace psychological demand (eg, perceived stress and depersonalization, *r* = 0.44). Factors influencing psychological resilience among men were suggested by significant positive correlations between positive affect (lower anxiety), mindfulness, and high workplace social support (eg, mindfulness and social support, *r* = 0.40). Overall, the state of flourishing among men did not appear to have a significant association with any of the other evaluated factors.

### End-User Experience

Overall response rates to the 2 open-ended questions in the survey were 36 participants (56%) (25 [67%] women) and 30 participants (47%) (22 [73%] women), respectively. For question 1a (“which well-being offerings work for you?”), responses included advance scheduling, ESRT, and support resources (wellness days, fatigue and mental health resources, stipends, and healthy workplace food). For Question 1b, (“which well-being offerings don’t work for you?”), all responses reflected scheduling challenges (conflicts, lack of coverage, no advance planning) that prevented utilization of well-being resources. For question 2, “What would you like to see offered…?”, responses included better scheduling, more widely-available ESRT, and more support, both practical (eg, fitness or gym memberships, additional wellness days, affordable day care) and social (eg, team-building and family-inclusive events, more mentoring).

Analysis of the focus group data led us to identify 4 emergent themes (Table 3). Demand was referred to as necessary and motivating in the form of a challenge (quotation 1) but was associated with dread and resentment when it hindered rewarding aspects of work (quotation 2) or was unequally shared by team members (quotation 3). Control, referred to variably as negative or positive, was associated with frustration and anger when it led to inefficiency (quotation 4) but was referred to as positive in the form of thoughtful and/or effective scheduling (quotation 5). Control in the form of choice was associated with greater work satisfaction (eg, residents’ choosing to extend hours for educational reasons; quotation 6). Support, both social (made possible by thoughtful and/or effective scheduling; quotation 7) and practical (in the form of well-being resources provided by the residency program; quotation 8), was referred to as essential but often absent or inaccessible. Affective regulation skills, in the form of reframing, acceptance, or perspective taking, were referred to as necessary and transformative (quotations 9 and 10), with multiple references to overwhelming negative emotions when such skills were absent (quotation 11).

**Table 3.** Themes and Quotations From Focus Group

Quotation No.	Participant ID	Exemplary quotation
**Demand**		
1	FGP-1W	“(Some) rotations are necessary evil. You need stress and fatigue to learn what you need to learn.”
2	FGP-2W	“You know that seeing the patient … always feels good, always makes things seem worth it. But [due to EMR burden] you feel dread, then you feel tremendous guilt. There’s no way out.”
3	FGP-3W	“Seeing NPs (and others) leave at 6pm you feel both resentful and guilty. Other groups are treated like humans, and it feels like crap to be the only group that’s not.”
**Control**		
4	FGP-3W	Having “no autonomy, [reiterating] consults, plans … for multiple people. [This is] totally inefficient and infuriating.”
5	FGP-4W	“At least 1 holiday off [really works]. Having the schedule 3 months in advance helps [maintain] social life.”
6	FGP-5M	“Lack of control … no idea of when you can or will leave—that is worse. It’s better when it’s a choice, [when you stay for] a good case, [something] educational.”
**Support**		
7	FGP-2W	“[PGY] 2/3s never see people. … More days are long, never have plans, [because] there’s no schedule. All the pressure from the day, the support you need, falls on the people at home. There’s a lot of strain.”
8	FGP-6M	“I needed [ESRT] more as a [PGY]2 and [PGY]3. Repeating it would have really helped. [As] a [PGY]3 you really need something. … Learn it as interns, build on it as a [PGY]2 and maybe more as a [PGY]3.”
Affective regulation skills		
9	FGP-7W	“We want to own our patients, to do the operation, to be in charge … resentment, guilt, dread, that’s on us. We have to make our own peace with [our choices and deciding] what’s ‘enough.’”
10	FGP-8M	“Maybe mid–third year, getting a new consult was no longer fun … It was a drag … I had to remind myself that a consult is a call for help. This was a [mental] shift I had to make to survive.”
11	FGP-3W	“I felt the rage for sure—simmering rage and acute rage, I felt it a lot. Because of your professional responsibility you always choose your patient first, you never choose yourself first. So, you begin to really feel resentful.”

Abbreviations: EMR, electronic medical record; FGP, focus group participant; ID, identification; M, men; NP, nurse practitioner; PGY, postgraduate year; W, woman.

## Discussion

The results of this mixed-methods study of well-being among surgical trainees at a single institution support 3 main findings. First, the prevalence of specific risk and resilience factors differs by gender identity; second, there are targetable individual characteristics and workplace elements that are associated with trainee well-being; and third, work quality and context, compared with work quantity, may be more associated with well-being.

Our first finding, that the prevalence of risk and resilience factors differed by gender identity, is supported by the significantly increased odds of high depersonalization among women trainees and of high inherent mindfulness among men trainees. Emotional exhaustion and depersonalization are increasingly recognized as representing different processes within burnout.^3^ In 1 of the few longitudinal causal studies of burnout in physicians,^40^ results suggest that depersonalization may be relatively protective against stress and anxiety, possibly through the mechanism of ego-defense. This corresponds with Maslach’s original work, in which the development of what she termed detached concern was interpreted as a protective mechanism, with depersonalization representing a dysfunctional extreme.^41^ Later studies have found that a high degree of concern for the patient, coupled with high detachment, can allow for emotional distance, which is protective regarding burnout development.^42^ This role for depersonalization is further supported by the Job Demand–Resources model of occupational strain,^43^ wherein women are particularly prone to disengagement and/or detachment when workplace stress is high and social support is poor.^36^ As such, provision of resources that support healthier coping skills (eg, available and accessible ESRT) and socialization (eg, mentoring, advance scheduling, and organized events) may be particularly important.

Relatedly, increased odds of higher mindfulness among men may be a marker of a local culture in which men feel greater ease. Although the CAMS-R instrument was designed to capture attention, present focus, awareness, and acceptance,^33,44^ these attributes are not exclusive to mindfulness-meditation training and can be found in individuals with resilience and healthy coping skills.^45^ Increased odds of high mindfulness tendencies in men may reflect a local culture in which men are more readily able to thrive. This possibility is further supported by the tendency for flourishing to be more prevalent in men (Table 2) and a growing body of work demonstrating greater mistreatment of women in surgical training nationally.^4,46^

Our second main finding, that there appear to be targetable elements that are associated with trainee well-being, is supported by the patterns and trends observed in correlations between individual risk and resilience factors and mindfulness, workplace social support, and workplace psychological demand. Overall, findings from both men and women reflect Broaden-and-Build theory,^13^ in that high positive affect (higher positive emotions) was correlated with higher mindfulness tendencies, while high negative affect (higher negative emotions) was correlated with higher burnout. These observations echo a strong body of theoretical work and empirical data suggesting reciprocity between positive affect and mindfulness and the ability of these internal states to promote resilience, meaning making, and higher life and work satisfaction.^47^ Findings from both genders also reflect Job Demand–Resource theory in that higher workplace psychological demand was correlated with psychological risk factors, and higher workplace social support was correlated with psychological resilience factors. Extensive work in occupational science demonstrates that job strain increases with greater psychological demand^43^ and is mitigated by greater social support, particularly among women.^36^

**Figure 2.**
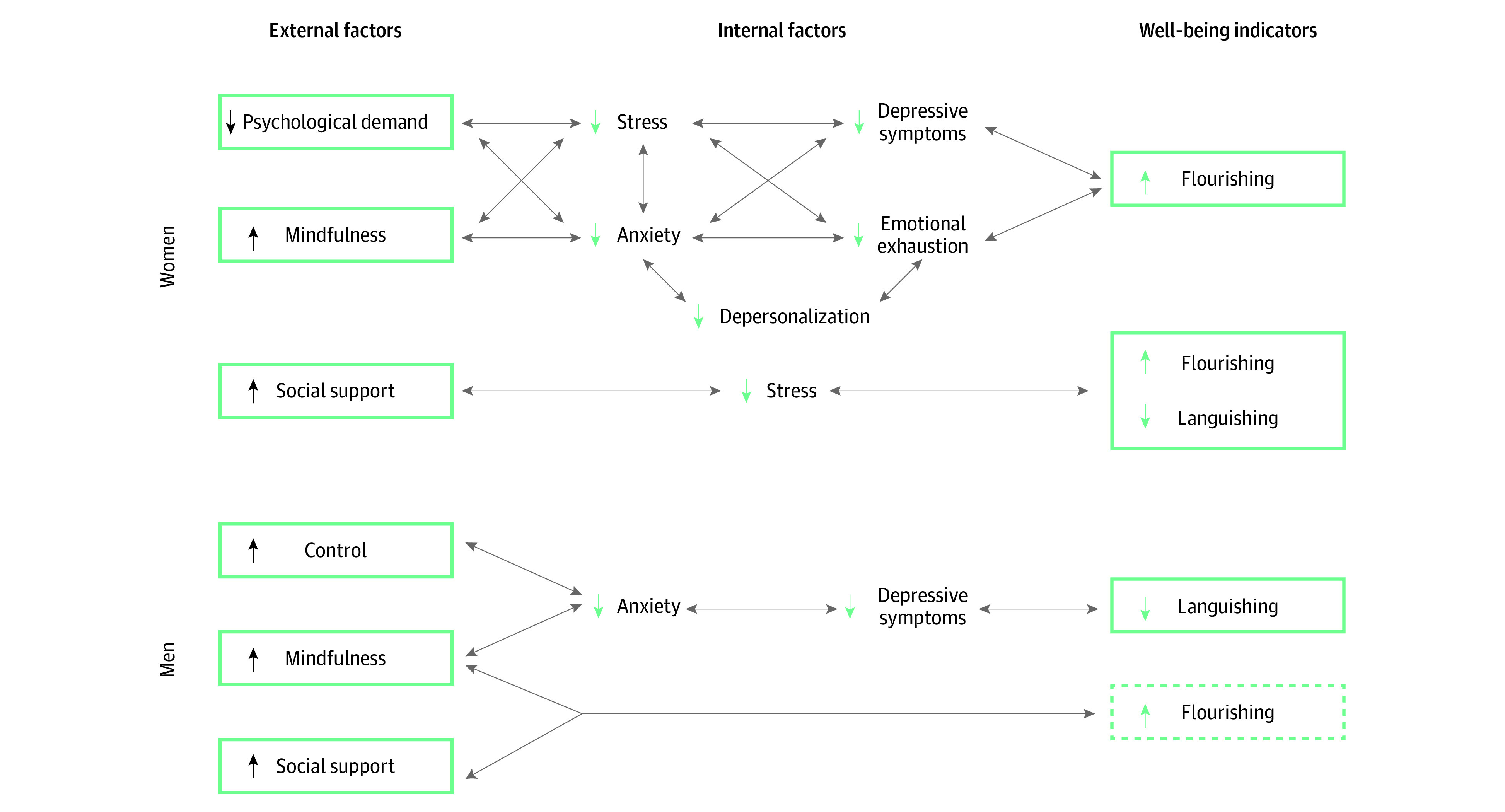
Conceptual Framework of Psychosocial External and Internal Factors Associated With Well-being in Men and Women Surgical Trainees Solid lines indicate statistically significant moderate or strong correlations; dashed lines indicate moderate or strong correlations.

Although correlation is not causation, this constellation of findings is important because it allows first-pass construction of a conceptual framework for how well-being may be affected (and potentially targeted) in the setting of surgical residency (Figure 2). Because of the cross-sectional nature of our study, these nascent models are not causal nor definitive, but they do allow us to begin a targeted approach to designing multilevel well-being initiatives. For example, mindfulness is a resilience factor that occurs naturally but can also be trained, as seen in our work with ESRT^19^ and elsewhere.^48^ Social support can be targeted to enhance resilience and mitigate burnout through formal mentorship,^22,49^ addressing mistreatment,^3,46,50^ or advance scheduling that facilitates social connections outside work. Psychological demand could be targeted in terms of work quality (eg, decreasing administrative burden^51^) rather than simply quantity (eg, work-hour restriction^52^). The previously described correlations and potential gender differences are intriguing but should be confirmed in future studies in light of our limited sample size and single-institution setting.

One gender-based finding worth noting is that none of the individual factors or workplace elements evaluated here appeared to have a significant correlation to flourishing in males. This is striking, because flourishing represents a more global conceptualization of well-being that encompasses social, emotional, and psychological aspects^7,34^ and allows us to monitor health, not just pathology. This metric accounts for individual variation in what constitutes well-being and recognizes that thriving is the goal even if achieved by various means. Additionally, flourishing has clinical relevance because it has been shown to mitigate the longitudinal development of depressive symptoms in large, multi-institutional studies of mixed specialty surgical trainees in PGY 1.^7^ Therefore, our findings suggest that additional factors should be evaluated in the interest of identifying the most effective well-being targets for men trainees. Increased control is a well-characterized way to mitigate job strain, particularly in men,^35^ making this a critical metric to evaluate in future work.

Our third finding, that work quality may be more strongly associated with resident well-being than work quantity, was supported by the findings of our focus group, which reflected the importance of context (Table 3). For example, results described demanding work as a challenging opportunity when demand is manageable but a source of resentment and distress when demand is inequitably shared or subsumes occupational rewards. Similarly, control in the form of self-determination was described as making additional work feel worthwhile, whereas it bred frustration in the context of inefficient hierarchical systems. Finally, our results reflected the potency of both social support and affective regulation skills in their ability to mitigate negative emotional influences on trainee work satisfaction. When social support was facilitated and affective regulation (ie, coping) skills were provided, trainees described the ability to navigate the intense emotional landscape of surgical training. These findings are supported by studies of job strain that show, for example, that workplace demand is not homogeneous and can promote or diminish work engagement when seen as a challenge vs a hindrance, respectively.^53^

### Limitations

While potentially provocative, our findings should be viewed in the context of several limitations. Specifically, while response rates and respondent demographic characteristics suggest representation of our population, our sample size is small owing to the fixed sized of our residency cohort. This should inspire caution in interpreting our findings, even those with statistical significance, pending confirmation in a larger multi-institutional study. Similarly, higher emotional exhaustion and alcohol abuse among women and higher flourishing among men had 95% CIs suggestive of potential statistical significance in the setting of adequate power, again underscoring the benefit of a larger, more definitive study.

## Conclusions

The findings of this study underscore the importance of accounting for individual experience, gender difference, workplace systems, and work quality in the design and assessment of well-being interventions. They highlight the value of training in affective regulation (eg, ESRT) and the synergy and necessity of using both individual and organizational-level elements to maximize well-being program effectiveness. Next steps involve conducting a larger multi-institutional study, using our findings to design strategies for targeting resources, and designing a multilevel longitudinal well-being curriculum tailored to each phase of surgical residency training.
